# Are You Sure? Confidence about the Satiating Capacity of a Food Affects Subsequent Food Intake

**DOI:** 10.3390/nu7075088

**Published:** 2015-06-24

**Authors:** Helgi B. Schiöth, Danielle Ferriday, Sarah R. Davies, Christian Benedict, Helena Elmståhl, Jeffrey M. Brunstrom, Pleunie S. Hogenkamp

**Affiliations:** 1 Department of Neuroscience, Uppsala University, Uppsala SE-751 24, Sweden; E-Mails: helgi.schioth@neuro.uu.se (H.B.S.); Christian.benedict@neuro.uu.se (C.B.); 2 Nutrition and Behaviour Unit, School of Experimental Psychology, University of Bristol, Bristol BS8 1TU, UK; E-Mails: Danielle.Ferriday@bristol.ac.uk (D.F.); sarah.r.davies@bristol.ac.uk (S.R.D.); Jeff.Brunstrom@bristol.ac.uk (J.M.B.); 3 Department of Food, Nutrition and Dietetics Uppsala University, Uppsala SE-751 24, Sweden; E-Mail: helena.elmstahl@ikv.uu.se

**Keywords:** satiety, satiation, expectations, compensation, overconsumption, expected satiation, confidence, energy density

## Abstract

Expectations about a food’s satiating capacity predict self-selected portion size, food intake and food choice. However, two individuals might have a similar expectation, but one might be extremely confident while the other might be guessing. It is unclear whether confidence about an expectation affects adjustments in energy intake at a subsequent meal. In a randomized cross-over design, 24 subjects participated in three separate breakfast sessions, and were served a low-energy-dense preload (53 kcal/100 g), a high-energy-dense preload (94 kcal/100 g), or no preload. Subjects received ambiguous information about the preload’s satiating capacity and rated how confident they were about their expected satiation before consuming the preload in its entirety. They were served an *ad libitum* test meal 30 min later. Confidence ratings were negatively associated with energy compensation after consuming the high-energy-dense preload (*r* = −0.61; *p* = 0.001). The same relationship was evident after consuming the low-energy-dense preload, but only after controlling for dietary restraint, hunger prior to, and liking of the test meal (*p* = 0.03). Our results suggest that confidence modifies short-term controls of food intake by affecting energy compensation. These results merit consideration because imprecise caloric compensation has been identified as a potential risk factor for a positive energy balance and weight gain.

## 1. Introduction

People often have clear expectations about the satiating capacity of a food. These expectations about fullness and satiety predict self-selected portion size, food intake and food choice [1,2,3]. After consumption, these expectations also appear to influence self-reported hunger and fullness. Indeed, several studies suggest that subtle modifications to the sensory characteristics of a food can change these expectations. In turn, this appears to have a marked effect on its effect on satiety [4,5,6,7] and on subsequent energy intake [7,8], as expectations can override physiological cues [9,10,11,12,13]. For example, manipulation of beliefs about the healthiness of a food affected subsequent intake [12,13]; and Crum *et al.* reported a steeper decline of the appetite-stimulating hormone ghrelin when participants consumed a milkshake that they believed was “indulgent” as compared with consuming the same milkshake that they believed was “sensible” [10]. There may be several factors contributing to this cognitively induced satiety, such as congruency between a believed satiating capacity and the actual satiating value (calorie content) of the food [3]. Another contributor to the satiating effect of a food or to decisions on the amount of energy consumed in a subsequent meal could be confidence about a specific satiety expectation. Two individuals might have a similar expectation about the satiating capacity of a food. However, one person might be extremely confident while the other might be very uncertain [14]. The aim of this experiment was to investigate whether confidence about the expected satiating capacity of a food modulates energy intake compensation. Subjects participated in three breakfast sessions, consuming a low energy-dense (LE) preload, a high energy-dense (HE) preload, or no preload. Subjects received ambiguous information about the preload’s satiating capacity: it was left unclear if they would receive a preload with a satiating agent (*i.e.*, the HE preload) or without this agent (*i.e.*, the LE preload). Subjects tasted a spoonful of the preload and rated their confidence about their expected satiation. They then consumed the preload in its entirety and were served an *ad libitum* test meal 30 min later. We measured energy intake compensation, *i.e.*, the extent to which adjustment in test meal intake “compensates” for the difference in energy content of consuming the preload.

## 2. Methods

### 2.1. Design and Subjects

Twenty-four healthy subjects (23 ± 2.9 years, M/F = 10/14; BMI = 22.8 ± 4.6 kg/m^2^; restraint score = 11.9 ± 4.0) used to eating breakfast regularly (≥5 times per week) participated in randomized cross-over experiment with three conditions. Exclusion criteria were: lack of appetite, following an energy-restricted diet or change in body weight ≥5 kg during the last two months; stomach or bowel diseases; diabetes; thyroid disease or any other endocrine disorder; hypersensitivity/allergy for the ingredients of the foods under study; smoking; and being a vegetarian. In separate breakfast sessions after an overnight fast, subjects were served a LE preload, a HE preloador no preload in a randomized order. Thirty minutes after the preload, they were served an *ad libitum* test meal and intake (kcal) was measured. The study procedures were explained in a meeting before the first session. In this meeting, the participants also provided written informed consent and completed the Three Factor Eating Questionnaire (TFEQ) [15]. Participants were unaware of the exact aim of the study, and were debriefed upon completion of the experiment. All procedures were evaluated by the Regional Ethic Review Board in Uppsala.

### 2.2. Test Foods

Subjects were served smoothies that had either a LE density (53 kcal/100 g) or a HE density (94 kcal/100 g) (see Table 1 for ingredients and sensory profile). Females were served 250 g portions and males were served 300 g. All preloads were consumed with a spoon. The LE and HE versions were matched for their sensory attributes, which was tested in a pilot study. Specifically, ten healthy young adults (M/F = 4/6; BMI = 25.7 ± 4.6 kg/m^2^; age = 27 ± 5.0) were unable to identify a difference between the preloads using a triangle test (*p* = 0.14). During this test, the subject was presented with one different and two similar samples of the preload, with the three samples presented at once and the possible combinations randomized across subjects. Subjects were instructed to taste the samples, to identify the odd one out and to record his/her answer. Participants in the main experiment noticed a difference when they conducted a triangle test at the end of the last session (*p* = 0.03). They did, however, not report significant differences between the LE and HE preload in the sensory ratings for any of the characteristics. The *ad libitum* test meal consisted of 16 triangles of fruit and nut bread (Fruktkusar, Fazer) with cream cheese (Cream Cheese nature l 9% fat, ICA). Each triangle with cream cheese weighed 23 g and contained 59 kcal. The triangles were presented *ad libitum* on a large plate, and participants ate and served themselves alone. They were informed that they could refill the plate if they wanted. Sixteen participants took advantage of this opportunity. All were served a glass of water (210 mL) with their meal.

**Table 1:** Ingredients, sensory profile and liking ratings (mean ± SD) of the preloads served in the experiment (*n* = 24).

Ingredients (g/100 g)	Low energy density (53 kcal/100 g)	High energy density (94 kcal/100 g)
Low-fat mild yogurt	33	-
High-fat mild yogurt	-	24 (23.8)
Water	27 (26.8)	27 (27.1)
Semi-skimmed milk	8.6	8.7
Banana	8.6	8.7
Frozen strawberries	8.6	8.7
Cream Cheese (9% fat)	8.6	5.8
Angel Delight strawberry flavor	1.7	1.7
Hartley’s Jelly strawberry flavor	1.3	1.3
Lemon juice	1.3	1.7
Benefiber powder	1.0	1.1
Stevia sweetener	0.6	0.2
Maltodextrin	-	11.3
**Sensory characteristics** *		
Sweet	75 ± 19	75 ± 17
Creamy	54 ± 21	50 ± 22
Thick	27 ± 25	25 ± 21
Filling	24 ± 16	25 ± 23
Liking	48 ± 24	44 ± 23

* Ratings obtained after the *ad libitum* meal. Participant did not report significant differences between the low energy and high energy preload for any of the characteristics.

### 2.3. Procedures

When the subjects were served the preload, they were told that “the ethical board wants the researchers to inform all participants about the non-commercial ingredients of the foods” and were shown a package with “a satiety agent and fiber blend that could be an ingredient of your food” (flour and sugar-mixture). They were then informed that “that it is unknown whether you have been served the food with the filling mixture or not” and that the researchers “also do not know which version of the food they consume, as the versions have the same sensory characteristics”. Subjects were then asked to taste the preload, to report which version of the preload they thought they had been served (with or without mixture), and to rate the confidence about their answer on a 100 mm visual analogue scale (VAS), anchored “not at all confident” and “extremely confident”. Participants then consumed the preload in its entirety. An *ad libitum* test meal was served 30 min after the preload.

A set of appetite and mood questions was included to detract subjects from the main aims of the study. Among these were questions measuring hunger and fullness (VAS, anchored “not at all” and “extremely”). Questions were completed on arrival, after consumption of the preload, just before and directly after the *ad libitum* test meal, and 1, 2, and 3 h after the *ad libitum* meal. To limit the possibility to “induce” restraint (potentially induced by paying attention to the preloads [16]), participants completed ratings on the sensory characteristics of the preload after the *ad libitum* meal. To do so, they rated sweetness, creaminess, thickness and fillingness, as well as whether they liked it, on a 100-mm VAS.

### 2.4. Data Analysis

Continuous variables are presented as means (±SD). Energy compensation (%) was calculated as [(*ad libitum* intake_no_ _preload_ − *ad libitum* intake_preload_)/energy content preload] × 100%. ANOVA (repeated measures) was used to test the effects of preload condition on *ad libitum* energy intake, and appetite ratings, and paired t-tests to test for differences in energy compensation and ratings of sensory attributes of the two preloads. Pearson’s correlation was used to assess associations between uncertainty scores and energy compensation. These associations were also controlled for possible differences in restraint, hunger ratings before the test meal, and liking of the test meal. Data were analyzed using SPSS software (SPSS Inc, Chicago, IL, USA). Results at a p-value of <0.05 were considered significantly different.

## 3. Results

**Figure 1:**
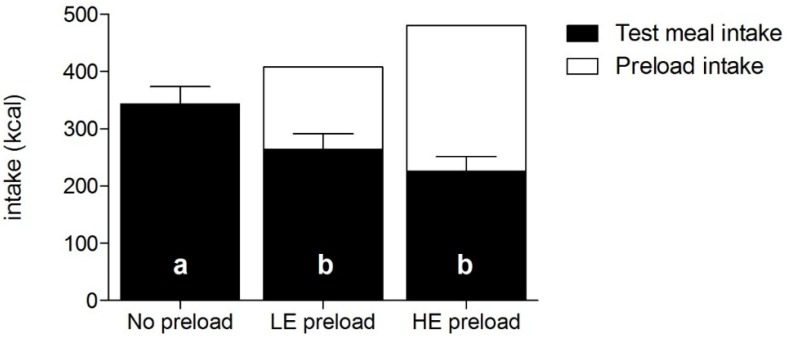
Mean intake (kcal ± SEM) of the fixed yogurt-based preload and of the *ad libitum* test meal consisting of sandwiches. a, b *Ad libitum* intake was greater when no preload was served (a) than following both the low-energy (LE) and the high-energy (HE) preload (b).

Energy intake compensation was similar following the LE (56% ± 1%) and HE preload (48% ± 1%, *p* = 0.62). In line, *ad libitum* test meal intake (kcal) depended on energy content of the preloads (*F* = 6.69, *p* = 0.001). Figure 1 shows energy intake of the test meal and the preload. Intake was greater when no preload was served (test meal intake: 343 ± 150 kcal) than following both the LE (test meal intake: 264 ± 134 kcal; *p* = 0.02) and the HE preload (test meal intake: 226 ± 124 kcal; *p* = 0.001). Test-meal intake after the LE preload did not differ significantly from intake after the HE preload (*p* = 0.13). These findings remained unchanged after controlling for restraint scores in these analyses. Total energy intake (*i.e.*, preload plus test meal) was higher in the HE-condition (480 ± 140 kcal) than in the LE-condition (407 ± 138 kcal) (*t* = −2.91, *p* < 0.01). For both the sessions with the LE and HE preloads, 19 of 24 participants thought they were served the preload with the added satiety agent. Confidence scores (*i.e.*, VAS-ratings that indicated how certain participants were about this answer) did not differ statistically between the conditions with the LE preload (mean = 48 ± 5 mm; median = 52 mm) and HE preload (mean = 54 ± 5; median = 60 mm) (*p* = 0.23). The scores were associated with energy compensation following the HE preload: participants who were less certain showed better compensation (*r* = −0.61; *p* = 0.001), *i.e.*, they compensate for more calories of the preload consumed recently by limiting kcal intake during the test meal. This association was not observed for the LE preload (*r* = −0.25, *p* = 0.24). When we included restraint scores, hunger ratings before the test meal, and liking ratings for the test meal sandwiches, uncertainty ratings were significantly associated with energy compensation following the HE preload (*F* = 10.76, *p* = 0.004), as well as the LE preload (*F* = 5.72, *p* = 0.027).

**Figure 2:**
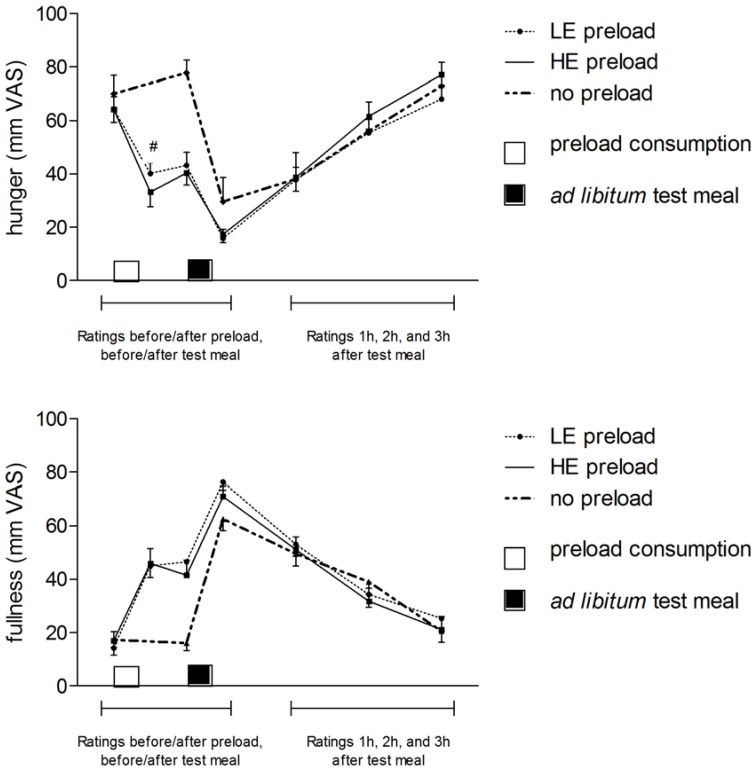
Mean values (mm VAS ± SEM) of self-reported hunger and fullness ratings when consuming no preload or the low-energy (LE) or high-energy (HE) preloads. The small blocks on the x-axis represent consumption of the preload (white block) and the ad libitum test meal (black block). # The difference in hunger ratings between LE and HE preload directly after consumption was borderline significant.

Hunger ratings (Figure 2) at arrival were similar across all conditions. Self-reported hunger ratings did not differ between sessions with the LE and HE preload on any of the time points, although the greater hunger that participants reported directly after consumption of the LE preload as compared to the HE preload was borderline significant (*t* = 1.84, *p* = 0.07). Self-reported hunger was greater in the no-preload condition as compared to both the LE and HE preload conditions until directly after the test meal. A similar––but contrasting––pattern was observed for fullness ratings. Confidence scores did not correlate with hunger ratings and fullness ratings.

## 4. Discussion

Our results showed that a lower confidence about the expected satiating capacity of a HE preload was associated with higher energy compensation. A similar, but weaker relationship was observed for LE preloads, which was only significant in the corrected model.

Our findings suggested that confidence is associated with less accurate energy compensation, which was in contrast with our expectations. A previous study suggested that uncertainty about post-ingestive effects of a test pizza meal that predicted less accurate compensation––although observed only in those with high scores on trait loss aversion [17]. These contrasting results may be explained by differences in familiarity of the test foods [14] (with familiar pizza *vs.* the test food in the current experiment that was consumed only once), differences in dietary variability (*i.e.*, consuming various versions of a type of food with different energy densities [17]) and/or by individual differences (with people who were less confident are so because they normally use the post-ingestive effects rather than expectations in order to regulate intake). We did not ask for the dietary variability of smoothies or dairy products among the participants, so we could not control for that. In the current study, the less confident participants could not rely on the information provided or on previous experience with the preloads, and may therefore have used the physiological cues for control of subsequent food intake––the only sure thing to rely on, which have improved their accuracy in energy intake compensation. Therefore, generalization of our results to daily life may be limited to experiences with novel foods and depend on individuals’ satiety responsiveness. The extent to which individuals pay attention to post-ingestive consequences over many meals may be limited, though, and imprecise caloric compensation has been identified as a potential risk factor for excess energy intake [18]. Moreover, based on the current findings we cannot conclude on the mechanisms that underlie the association between expected-satiation confidence and food intake control. Future studies could systematically vary the confidence to give further insight in the relation between confidence and energy compensation.

As it has been observed that energy compensation is poorer for preloads that are denser [18], we included a LE and HE preload to assess if confidence would affect energy compensation differently for these preloads. Energy compensation percentages did not differ between the LE and HE conditions. The energy density of the HE preloads (94 kcal/100 g) was indeed higher than that of the LE preload, but in absolute sense the HE preload might still be considered as a low(er)-energy-dense food. Still, the increase in the energy density resulted in a higher total energy intake in the HE condition, while hunger ratings were similar to the LE condition. This again suggests that decreasing energy density is a fruitful strategy to decrease overconsumption [19]. In addition to larger energy differences, it would have been good if preload recipes would ensure that the proportion of energy provided by the different macronutrients was similar for both preloads.

Only five participants wrongly identified the HE preload (*i.e.*, reporting not having the added satiety agent) when asked to state which version of the preload they received after tasting a single spoonful, and five correctly identified the LE preload. This limited the power to assess whether the belief itself or the confidence about this belief was a more important determinant of subsequent intake. Participants of the pilot-study did not detect a difference between the LE and HE preloads when conducting the triangle-test, while the statistics of the main study showed a significant difference between the LE and HE samples: about half of the participants was able to identify the odd sample out. We did not, however, observe differences between ratings of the sensory attributes of the LE and HE preloads; and almost 80% of the participants expected the preload to be filling in both the LE and HE condition. It may therefore be reasonable to assume that the sensory characteristics limited the possibility to predict the post-ingestive consequences of the preloads, which could explain the absence of differences in energy compensation between the LE and HE preload. Variation in, e.g. the sensory ratings of the current sample, was, however, high, as these subjects were not trained to perform a sensory analysis food and ratings were conducted at the end of the test session. First of all, a larger number of subjects would have increased the possibility to generalize of the results. Second, an additional “standard” or control condition in which participants are confident about the fillingness of the preload, based on e.g. true and reliable information, would have given further insight in the relation between confidence and energy compensation. Finally, a measure of expected satiation [20] directly after of tasting the food would have allowed us to analyze if expected satiation and/or confidence ratings would predict compensation scores. Future studies should consider including these measures and conditions as well as a larger sample size, to further conclude on the role of confidence in our eating behavior.

Our findings nevertheless suggest that confidence modulates energy compensation, albeit in a laboratory environment when preloads are served with similar sensory characteristics. High confidence was associated with less accurate energy compensation in a single meal. Participants who were less confident under these conditions may have relied more on physiological cues.

## 5. Conclusions

Our results suggest that confidence modifies short-term controls of food intake by affecting energy compensation. These results merit consideration because imprecise caloric compensation has been identified as a potential risk factor for a positive energy balance and weight gain.

## References

[1] Brunstrom J.M., Rogers P.J. (2009). How many calories are on our plate? Expected fullness, not liking, determines meal-size selection. Obesity.

[2] Wilkinson L.L., Hinton E.C., Fay S.H., Ferriday D., Rogers P.J., Brunstrom J.M. (2012). Computer-based assessments of expected satiety predict behavioural measures of portion-size selection and food intake. Appetite.

[3] Bilman E. (2014). Claiming Satiety: Consumer Perception, Interpretation and Subsequent Food Intake. Doctoral Dissertation.

[4] Hogenkamp P.S., Stafleu A., Mars M., Brunstrom J.M., de Graaf C. (2011). Texture, not flavor, determines expected satiation of dairy products. Appetite.

[5] McCrickerd K., Chambers L., Brunstrom J., Yeomans M. (2012). Subtle changes in the flavour and texture of a drink enhance expectations of satiety. Flavour.

[6] Yeomans M.R., Chambers L. (2011). Satiety-relevant sensory qualities enhance the satiating effects of mixed carbohydrate-protein preloads. Am. J. Clin. Nutr..

[7] Yeomans M., McCrickerd K., Brunstrom J.M., Chambers L. (2013). Effects of repeated consumption on sensory-enhanced satiety. Brit. J. Nutr..

[8] Hogenkamp P. (2014). The effect of sensory-nutrient congruency on food intake after repeated exposure: Do texture and/or energy density matter?. Physiol. Behav..

[9] Cassady B.A., Considine R.V., Mattes R.D. (2012). Beverage consumption, appetite, and energy intake: What did you expect?. Am. J. Clin. Nutr..

[10] Crum A.J., Corbin W.R., Brownell K.D., Salovey P. (2011). Mind over milkshakes: Mindsets, not just nutrients, determine ghrelin response. Health Psychol..

[11] Hogenkamp P.S., Cedernaes J., Chapman C.D., Vogel H., Hjorth O.C., Zarei S., Lundberg L.S., Brooks S.J., Dickson S.L., Benedict C. (2013). Calorie anticipation alters food intake after low-caloric not high-caloric preloads. Obesity.

[12] Provencher V., Polivy J., Herman C.P. (2009). Perceived healthiness of food. If it’s healthy, you can eat more!. Appetite.

[13] Faulkner F.P., Pourshahidi L.K., Wallace J.M., Kerr M.A., McCaffrey T.A., Livingstone M.B. (2014). Perceived “healthiness” of foods can influence consumers’ estimations of energy density and appropriate portion size. Int. J. Obes..

[14] Brunstrom J.M., Shakeshaft N.G., Alexander E. (2010). Familiarity changes expectations about fullness. Appetite.

[15] Cappelleri J., Bushmakin A., Gerber R., Leidy N., Sexton C., Lowe M., Karlsson J. (2009). Psychometric analysis of the Three-Factor Eating Questionnaire-R21: Results from a large diverse sample of obese and non-obese participants. Int. J. Obes..

[16] Robinson E., Aveyard P., Daley A., Jolly K., Lewis A., Lycett D., Higgs S. (2013). Eating attentively: A systematic review and meta-analysis of the effect of food intake memory and awareness on eating. Am. J. Clin. Nutr..

[17] Hardman C., Ferriday D., Kyle L., Rogers P.J., Brunstrom J.M. (2015). So many brands and varieties to choose from: Does this compromise the control of food intake in humans?. PLoS ONE.

[18] Almiron-Roig E., Palla L., Guest K., Ricchiuti C., Vint N., Jebb S.A., Drewnowski A. (2013). Factors that determine energy compensation: A systematic review of preload studies. Nutr. Rev..

[19] Rolls B.J. (2009). The relationship between dietary energy density and energy intake. Physiol. Behav..

[20] Brunstrom J.M., Shakeshaft N.G., Scott-Samuel N.E. (2008). Measuring “expected satiety” in a range of common foods using a method of constant stimuli. Appetite.

